# Navigated Intraoperative Ultrasound in Functionally Eloquent Tumour Resection: A Descriptive Single-Centre Series Within the Modern Multimodal Neuro-Oncology Surgery Workflow

**DOI:** 10.3390/jpm16100516

**Published:** 2026-10-02

**Authors:** Feras Fayez, Ahmed B. Abougamil, Nida Kalyal, Sarah Hatch, Qusai Al Banna, Sandie Eiras, Prajwal Ghimire, Francesco Marchi, Ali Elhag, Yasir A. Chowdhury, Richard Gullan, Francesco Vergani, Keyoumars Ashkan, Ranjeev Bhangoo, José Pedro Lavrador

**Affiliations:** 1 Department of Neurosurgery, King’s College Hospital NHS Foundation Trust, Denmark Hill, London SE5 9RS, UK; ahmed.abougamil@nhs.net (A.B.A.); nida.kalyal@nhs.net (N.K.); sarah.hatch3@nhs.net (S.H.); qusai.albanna@nhs.net (Q.A.B.); prajwal.ghimire@nhs.net (P.G.); francesco.marchi@nhs.net (F.M.); yasir.chowdhury2@nhs.net (Y.A.C.); richardgullan@nhs.net (R.G.); francesco.vergani@nhs.net (F.V.); k.ashkan@nhs.net (K.A.); ranj.bhangoo@nhs.net (R.B.); josepedro.lavrador@nhs.net (J.P.L.); 2 Victor Horsley Department of Neurosurgery, The National Hospital for Neurology and Neurosurgery, Queen Square, London WC1N 3BG, UK; sandie.eiras@nhs.net

**Keywords:** navigated intraoperative ultrasound, brain shift, glioma, brain metastasis, extent of resection, onco-functional surgery, neuro-oncology

## Abstract

**Background/Objectives:** Navigated intraoperative ultrasound (N-ioUS) compensates for brain shift, but its place within a multimodal workflow alongside neuronavigation, 5-aminolevulinic acid (5-ALA) fluorescence and neurophysiological monitoring is not well defined. We describe functional and surgical outcomes in a single-centre series of functional-eloquent tumours resected with N-ioUS within such a workflow. **Methods:** Retrospective descriptive cohort study, reported per STROBE, of 104 consecutive patients who underwent N-ioUS-assisted resection of functionally eloquent intracranial tumours between 2011 and 2023. N-ioUS was an inclusion criterion, so no comparison group was available; the same-institution 5-ALA cohorts are cited as context only. **Results:** The cohort comprised WHO grade 4 gliomas (*n* = 42), WHO grade 2–3 gliomas (*n* = 33), metastases (*n* = 21) and other tumours (*n* = 8); 5-ALA was used in 74.0% and neurophysiological monitoring in 94.2%. No residual tumour was described in 22.5% of grade 4 gliomas, 53.3% of grade 2–3 gliomas and 73.7% of metastases with a documented extent of resection. Among patients at risk with documented postoperative status, a new motor deficit occurred in 4/16 (25.0%) grade 4, 4/21 (19.0%) grade 2–3 and 1/7 (14.3%) metastases patients. Median overall survival was 12.7 months for grade 4 gliomas and 22.2 months for metastases and was not reached for grade 2–3 gliomas; within grade 4 gliomas, no difference by extent of resection was detected in an underpowered exploratory comparison (log-rank *p* = 0.593). **Conclusions:** In this single-arm series the resection endpoint in eloquent tumours was set by functional rather than anatomical boundaries, and rates of complete resection and new deficit were in keeping with that constraint. The design permits no inference about the independent effect of N-ioUS, whose contribution may be greatest where the endpoint is anatomical, as in metastases, and requires prospective controlled evaluation.

## 1. Introduction

The introduction of advanced imaging technologies such as magnetic resonance imaging (MRI) and computed tomography (CT) has significantly enhanced precision in neurosurgery, particularly in the resection of complex intracranial tumours [1,2,3,4]. Advanced derivative techniques, including diffusion tensor imaging (DTI), tractography, angiographic sequences, and functional neuroimaging, have substantially improved surgical planning and risk stratification [1,2,3]. However, despite these advances, preoperative imaging remains fundamentally static and cannot fully account for the dynamic intraoperative changes that occur during cranial surgery.

One of the most important of these phenomena is brain shift, which occurs secondary to cerebrospinal fluid drainage, tumour debulking, gravity, osmotic changes, and patient positioning, resulting in progressive displacement of intracranial structures throughout surgery [5,6]. In addition, intraoperative brain deformation further reduces the reliability of preoperative image guidance.

Two-dimensional intraoperative ultrasound (ioUS) has long been recognised as a valuable tool in neurosurgery because of its ability to provide immediate real-time imaging during surgery [5,7]. Navigated intraoperative ultrasound (N-ioUS), which integrates ultrasound imaging with preoperative MRI or CT-based neuronavigation, enables real-time image fusion and dynamic intraoperative updating of anatomical relationships [8]. This is particularly valuable for compensating for brain shift and improving surgical orientation during tumour resection [5,6,9].

Despite the increasing adoption of intraoperative MRI (iMRI) and intraoperative CT (iCT), intraoperative ultrasound remains highly relevant because of its accessibility, lower cost, portability, and ability to provide continuous real-time imaging without interrupting operative workflow [10,11,12]. Unlike iMRI, which requires substantial infrastructure and prolonged acquisition times, N-ioUS can be repeatedly employed throughout surgery with minimal disruption and without the need for dedicated operating theatre redesign [7,13]. Beyond greyscale B-mode imaging, ioUS also supports more advanced intraoperative applications. Real-time ultrasound guidance permits accurate cannulation of deep-seated cystic lesions so that a catheter, tubular retractor or endoscope can be directed into the cavity along a trajectory that is confirmed as the instrument advances rather than inferred from preoperative imaging [14,15]. Ultrasound elastography adds a measure of tissue stiffness to the B-mode image, and because tumour tissue commonly differs in stiffness from the surrounding brain, it can help to define the lesion boundary and to inform where a transsulcal corridor is docked [16,17].

Neuro-oncology resections that obey the onco-functional principle are becoming increasingly complex. The emergence of adjunctive technologies, including indocyanine green (ICG) fluorescence and 5-aminolevulinic acid (5-ALA), has further transformed neuro-oncological surgery, improved visualisation of vascular structures and perfusion and extended resection beyond the identifiable anatomical limits. Similarly, techniques such as transcranial magnetic stimulation (TMS), diffusion tensor imaging, and functional MRI (fMRI) have become increasingly important for functional preservation during surgery [1,18,19,20,21]. Finally, modern neuro-oncology surgery increasingly recognises that the endpoint of resection is determined not solely by anatomical tumour boundaries but by functional limitations identified through intraoperative neurophysiological monitoring (IONM), cortical mapping, and subcortical stimulation, including where supramaximal resection is intended [21,22].

In functionally eloquent tumours, these advances create a specific tension. Extending resection towards the radiological margin increases the probability of injuring cortical or subcortical structures whose function cannot be recovered, while stopping at the functional border leaves tumour behind that would be resectable on anatomical grounds alone. Reported rates of new postoperative neurological deficit after resection of tumours involving or abutting eloquent white matter tracts are substantially higher than for non-eloquent lesions, and although most such deficits improve, a proportion remain permanent [23,24]. The extent of resection achievable in this setting is therefore constrained by function rather than by image quality, and any imaging adjunct intended to improve resection must be judged against that constraint.

In this retrospective cohort study, we describe a single-centre series of patients who underwent resection of functionally eloquent intracranial tumours with navigated intraoperative ultrasound between 2011 and 2023, and consider the place of N-ioUS within a contemporary multimodal neuro-oncology surgical workflow. Because N-ioUS was used in every patient, this study is descriptive and does not test the effect of N-ioUS against an alternative.

## 2. Materials and Methods

This study was conducted per the STROBE guidelines for cohort studies. The STROBE checklist is provided in the Supplementary Materials (File S1). We performed a retrospective descriptive cohort analysis encompassing all patients who underwent tumour resection using navigated intraoperative ultrasound (N-ioUS) at our institution between 2011 and 2023. The study included patients of all age groups referred by the neuro-oncology multidisciplinary team (MDT). The indication for the use of N-ioUS was at the discretion of the surgical team responsible for the treatment. Because use of N-ioUS was an inclusion criterion, every patient received the intervention of interest, and no concurrent comparison group was constructed; the reasons are set out in the Limitations.

Only patients with tumours in functionally eloquent locations were included. Eloquence was defined a priori as a lesion-to-tract distance of less than 10 mm from an eloquent white matter tract, most commonly the corticospinal tract or the arcuate fasciculus, on preoperative tractography, or radiological involvement of the basal ganglia, internal capsule, insula, or cortex with an established motor or language function. This threshold follows the lesion-to-tract and cavity-to-tract distances previously validated at our institution as predictors of postoperative motor deficit [24,25,26].

To identify eligible cases, we systematically screened the electronic patient records at our centre using the keywords “ioUS,” “ultrasound,” “USS,” and “Intraoperative Navigation” within all neuro-oncology notes. Records were then reviewed against the eloquence criteria above. All resection approaches were included in the analysis, with no exclusions based on the method of application of ioUS.

Data collection focused on patient demographics (including age at the time of surgery, sex, type of surgery, adjuvant therapy, lesion location and preoperative neurological function); methods of surgical mapping, including the use of intraoperative neuromonitoring (IONM), navigation methodology, surgical adjuncts (endoscope, exoscope, ultrasound, 5-ALA, ICG, augmented reality); preoperative mapping techniques (tractography, TMS, functional MRI); and outcome variables such as postoperative neurological function, the number of days to discharge, extent of resection, post-op complications, histological findings and adjuvant therapy. Neurological status was assessed by the surgical team immediately on emergence from anaesthesia, at the daily ward round until discharge, and at each subsequent clinic appointment, the frequency of which varied with the patient and the pathology. Motor deficit, visual deficit and symptoms (headache, dizziness, collapse or seizures) were recorded in the database as present or absent before surgery and after surgery; the database holds a single postoperative entry per patient without a time point, so a deficit recorded after surgery cannot be assigned to the immediate postoperative period, to discharge or to clinic review. A new deficit was defined as a deficit recorded after surgery in a patient in whom it was absent before surgery, and its rate is reported among patients at risk, that is, those without the deficit before surgery and with documented postoperative status. Persistence of a preexisting deficit is reported separately. Because deficits were recorded as present or absent rather than graded, worsening of a preexisting deficit could not be distinguished from its persistence. Postoperative neurological status was undocumented in 9 of the 104 patients; these patients were excluded from the denominator of the postoperative proportions rather than assumed to be free of deficit.

### 2.1. Navigated Intraoperative Ultrasound and Surgical Adjuncts

**Figure 1:**
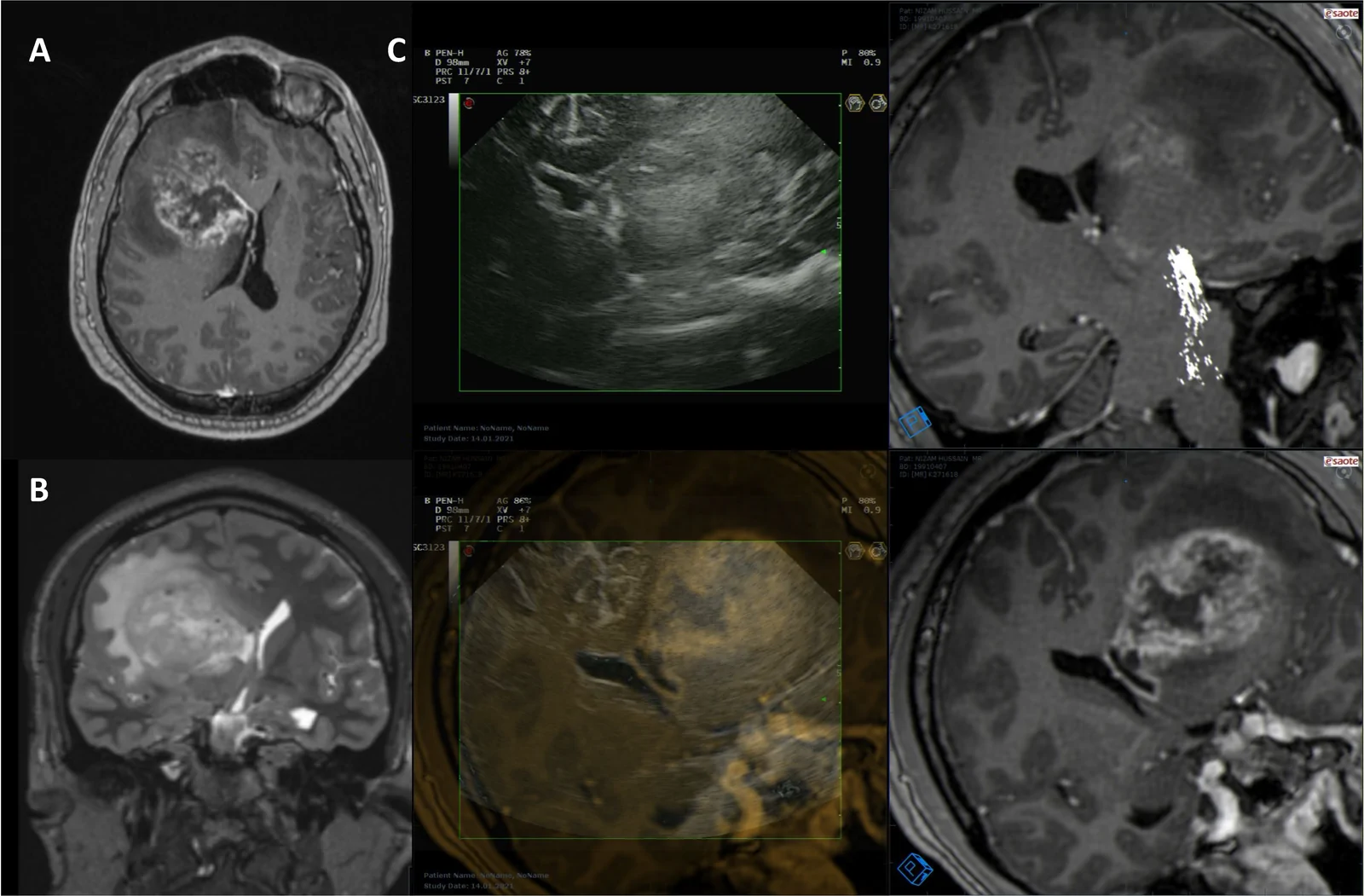
Illustrative case. Deep-seated periventricular WHO grade 4 glioblastoma, IDH wild-type. Axial T1-weighted image with gadolinium (**A**) and coronal T2-weighted image (**B**). Navigated intraoperative ultrasound (**C**) was used to assist the intraoperative assessment of the extent of resection, with the B-mode image and the co-registered preoperative magnetic resonance image displayed separately and in overlay. The figure illustrates the display used in this series and is not a quantitative assessment of registration accuracy or residual tumour.

**Figure 2:**
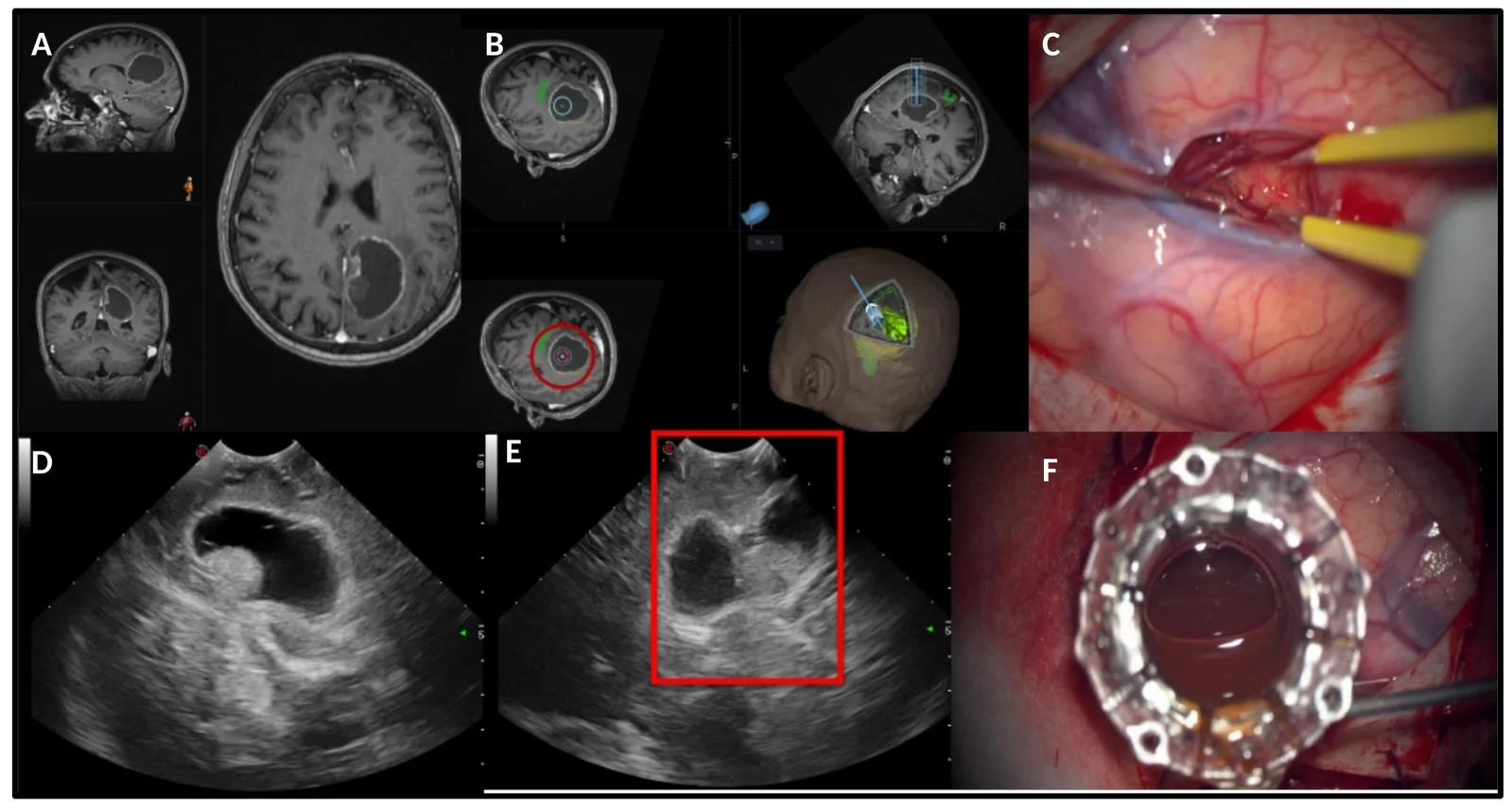
Illustrative case. Deep-seated cystic metastases. Axial, coronal and sagittal T1-weighted images with gadolinium (**A**). Preoperative tractography of the corticospinal tract, integrated with neuronavigation, informed the definition of a safe entry zone for a minimally invasive parafascicular approach (**B**). Sulcal dissection was performed (**C**) and the tubular retractor was advanced to the lesion (**F**). Navigated intraoperative ultrasound with the probe in a paratubular position allowed the cannulation to be carried out under direct visualisation (**D**,**E**); the red square marks the tubular retractor entering the cystic cavity. A tubular retractor was documented in 9 of the 104 index operations.

**Figure 3:**
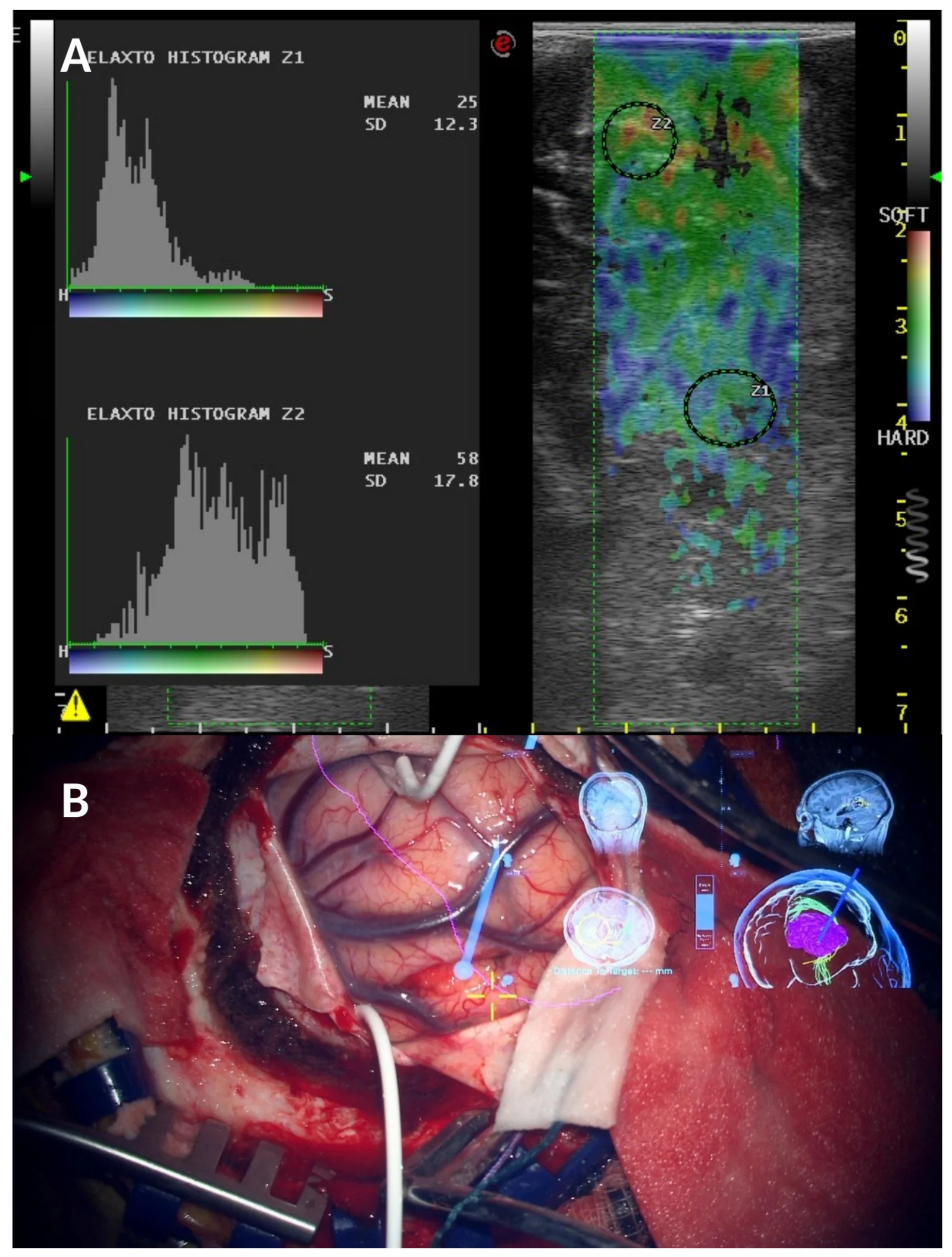
Illustrative case. Strain elastography (**A**) was used to assess the relative stiffness of the tumour tissue before a transsulcal approach to a deep-seated lesion. Two regions of interest were sampled on the colour-coded elastogram, with mean strain values of 25 (Z1) and 58 (Z2), which informed the docking position of the tubular retractor (**B**). Strain values are relative, unitless indices from a single case; they were not correlated with histology and are shown to illustrate the technique rather than as a validated measure of the tumour boundary. Strain elastography was documented in 5 of the 104 index operations.

Ultrasound was performed with a MyLab Eight eXP system (Esaote SpA, Genoa, Italy) and an SC3123 microconvex transducer (nominal bandwidth 6 to 10 MHz). The probe was tracked by, and co-registered to, the neuronavigation system (StealthStation with optical tracking or AxiEM electromagnetic tracking; Medtronic, Minneapolis, MN, USA), so that real-time two-dimensional B-mode images were displayed alongside, and fused with, the reformatted preoperative MRI in the same plane (Figure 1). Imaging was two-dimensional navigated B-mode ultrasound; three-dimensional volume acquisition and reconstruction were not used. The use of ioUS was not limited to the end of the resection as a check of the anatomical, oncological and functional boundaries. It was used repeatedly from dural and cortical exposure until the end of the resection, so that each successive image of the cavity and of the tumour or residual could be interpreted against the baseline acquired on the intact brain and against the intraoperative findings, including the vascular anatomy, the ventricular anatomy, the pial and arachnoid surfaces, and the functional boundaries defined by mapping. The number and timing of acquisitions were not recorded prospectively. Paratubular scanning during minimally invasive parafascicular approaches (Figure 2) and strain elastography (Figure 3) were used in selected cases at the discretion of the operating surgeon; contrast-enhanced ultrasound was not used. Operations were performed under 9 consultant neurosurgeons, four of whom accounted for 96 of the 104 procedures; scanning and interpretation were performed by the operating consultant neurosurgeon. Use of each ultrasound modality was ascertained from the operation title and from the structured adjunct fields of the departmental database and is summarised in the Results; because operation titles record adjuncts inconsistently, these counts are likely to understate use. Because ioUS was integrated throughout the resection rather than deployed as a single end-of-resection check, the number of occasions on which it changed surgical strategy was not recorded (see Limitations). 5-ALA (Gliolan; photonamic GmbH, Pinneberg, Germany) was administered orally before induction according to the licensed protocol. In tumours that were radiologically lower grade, including those subsequently confirmed on histology as WHO grade 2–3 glioma, 5-ALA was administered to identify possible foci of anaplastic transformation; 5-ALA fluorescence can localise in diffusely infiltrating gliomas without significant contrast enhancement [27,28]; this is particularly relevant where heterogeneity of diffusion restriction within the tumour suggested a higher-grade component, since focal reduction of the apparent diffusion coefficient in WHO grade 2 glioma is associated with, and may precede, malignant transformation [29,30]. This was in keeping with the practice of the unit during the study period [31]; the final diagnosis was assigned on integrated histopathology.

### 2.2. Extent of Resection

Extent of resection (EoR) was categorised following the recommendations of Karschnia et al. [32], using the categories supramaximal resection, gross total resection, near total resection, subtotal resection and partial resection, which were subsequently formalised by the RANO resect group [33,34]. Volumetric segmentation of pre- and postoperative MRI was not performed, so the category was assigned from the operative note and the postoperative radiology report and recorded as not documented where neither source recorded it. Where the two sources disagreed, or the entry was qualified as uncertain, the lower category was taken. Because the RANO resect classes are defined by residual tumour volume, they were not applied and no class labels are used. Results are presented as no residual tumour described (supramaximal or gross total resection) versus residual tumour described (near total, subtotal or partial resection), with percentages calculated among the patients in whom extent of resection was documented. For contrast-enhancing tumours this refers to residual contrast-enhancing tumour; for WHO grade 2–3 gliomas, which are largely non-enhancing, it refers to residual tumour on T2-weighted or FLAIR imaging as described in the radiology report. Patients with no documented extent of resection are reported separately and were excluded from the resection comparison.

### 2.3. Statistical Analysis

Statistical analyses were conducted using R (version 4.3.2; The R Foundation for Statistical Computing) and RStudio (version 2023.12.0+369). Continuous variables were summarised as means with standard deviations (SD) and categorical variables as frequencies and percentages. The denominator is stated for every proportion; patients with an undocumented value were excluded from the denominator for that variable rather than assumed to be negative. Vital status and date of death were ascertained from the clinical record at the final follow-up update. Overall survival was estimated using the Kaplan–Meier method, measured from the date of surgery to death or, for patients who remained alive, to the date of last follow-up, at which point observations were censored; median follow-up was estimated by the reverse Kaplan–Meier method. Patients with a missing or invalid survival time were excluded from the survival analysis, which therefore included 100 of 104 patients. As a single exploratory analysis, overall survival by extent of resection within the WHO grade 4 subgroup was compared using the log-rank test, with statistical significance defined as *p* < 0.05. Given the cohort heterogeneity and the small subgroup sizes, no other inferential testing was performed; survival by IDH status within the WHO grade 4 subgroup is reported descriptively without formal comparison, and all survival findings are regarded as exploratory and hypothesis-generating. No comparison between this cohort and the previously published same-institution cohorts was tested; those cohorts are cited for context only.

## 3. Results

### 3.1. Overall Cohort

A total of 104 patients underwent intracranial tumour resection using N-ioUS. The cohort was predominantly male (57.7%) with a mean age of 51.8 years (SD ± 15.5). The most common histological subtype was WHO grade 4 glioma (40.4%), of which 36 carried a diagnosis of glioblastoma; one was recorded as glioblastoma IDH-mutant, which under the fifth edition of the WHO classification corresponds to astrocytoma IDH-mutant CNS WHO grade 4; two as WHO grade 4 astrocytoma; two as high-grade glioma without a definitive entity; and one as anaplastic oligodendroglioma recorded as CNS WHO grade 4, a combination that does not exist in the current classification and could not be resolved because 1p/19q codeletion was not recorded. This case was retained in the grade 4 subgroup as reported; excluding it would change the grade 4 median overall survival from 12.7 to 12.2 months and survival at 12 and 24 months from 56.1% and 25.1% to 55.0% and 23.1%. WHO grade 4 glioma was followed by WHO grade 2–3 glioma (31.7%), metastatic tumours (20.2%), and other tumour types, including ependymoma, haemangioblastoma and WHO grade 1 glioma. Baseline demographics, tumour characteristics and tumour location are summarised in Table 1.

Before surgery, a motor deficit was recorded in 49 of 104 patients (47.1%), symptoms comprising headache, seizures, dizziness or collapse in 78 (75.0%) and a visual deficit in 16 (15.4%). Postoperative neurological status was documented in 95 of 104 patients (91.3%) and undocumented in 9 (3 grade 4, 5 grade 2–3 and 1 metastases). Among these 95 patients, a motor deficit was present after surgery in 28 (29.5%), symptoms in 6 (6.3%) and a visual deficit in 10 (10.5%). Among the 49 patients without a preoperative motor deficit and with documented postoperative status, 10 (20.4%) developed a new motor deficit. Of the 49 patients with a preoperative motor deficit, postoperative status was documented in 46: the deficit improved or resolved in 28 (60.9%) and persisted in 18 (39.1%); because motor function was recorded as present or absent, worsening within the persistent group could not be quantified. Of the 78 patients with preoperative symptoms, postoperative status was documented in 70, and symptoms had resolved in 65 (92.9%); one patient without preoperative symptoms had new symptoms after surgery. Of the 16 patients with a preoperative visual deficit, 9 improved or resolved and 7 persisted, and 3 of 79 patients at risk (3.8%) developed a new visual deficit. Neurological status before and after surgery for the whole cohort is summarised in Table 2.

The mean length of hospital stay across the cohort was 9.6 days (SD ± 11.5), recorded in 100 of 104 patients. Among the three main tumour subtypes, patients with metastatic disease had the longest mean stay (12.1 ± 15.7 days), and those with WHO grade 2–3 gliomas had the shortest (6.6 ± 3.1 days); the small other subgroup had a mean stay of 13.5 ± 19.0 days. The standard deviations exceeded the means in every subgroup except WHO grade 2–3 glioma.

### 3.2. Surgical Adjuncts and Adjuvant Therapy

All patients underwent surgery using N-ioUS. Surgical adjuncts and extent of resection are summarised by tumour subtype in Table 3, and neurological outcome, complications and oncological treatment in Table 4. Conventional neuronavigation was documented in addition to N-ioUS in 94 of 104 cases (90.4%), comprising Medtronic optical navigation in 82 patients and electromagnetic AxiEM navigation in 12. Navigation was registered to the preoperative volumetric MRI before draping, and accuracy was verified against anatomical landmarks prior to dural opening; the ultrasound probe was then co-registered to the navigation system to allow fusion of real-time ultrasound with the preoperative dataset. Intraoperative neurophysiological monitoring was used in 98 of 104 cases (94.2%), comprising 40/42 WHO grade 4 gliomas, 32/33 WHO grade 2–3 gliomas, 19/21 metastases and 7/8 other tumours. Tractography was used in 36.5% of surgeries (38/104), TMS in 22.1% (23/104) and functional MRI in 6.7% (7/104). Fluorescence-guided surgery with 5-aminolevulinic acid (5-ALA) was used in 74.0% of procedures (77/104), comprising 40/42 WHO grade 4 gliomas, 26/33 WHO grade 2–3 gliomas, 8/21 metastases and 3/8 other tumours. Indocyanine green (ICG) fluorescence was used in 12.5% of surgeries (13/104: 7/42 grade 4, 5/33 grade 2–3, 1/21 metastases), and augmented reality was integrated in 9.6% of cases (10/104: 4/42 grade 4, 4/33 grade 2–3, 2/21 metastases) [35,36,37,38,39,40]. The specific ultrasound modalities used are summarised in Table 5. Ultrasound was named in the operation title in 69 of 104 index operations (66.3%), and fusion with the navigated MRI dataset was named explicitly in 19. Strain elastography was named in 5 index operations (2 grade 4, 1 grade 2–3 and 2 metastases) and in 2 further reoperations (one grade 4, one grade 2–3); contrast-enhanced ultrasound was not used. A tubular retractor or transsulcal parafascicular approach was documented in 9 index operations (7 grade 4, 1 metastases and 1 other) and an endoscope in 5. Thirty-six index operations (34.6%) were performed awake or asleep-awake-asleep (13 grade 4, 21 grade 2–3 and 2 metastases). Because operation titles record adjuncts inconsistently, these figures are likely to understate actual use.

Documented postoperative oncological treatment was recorded in 68 of 104 patients (65.4%), comprising 35 of 42 WHO grade 4 gliomas (83.3%), 17 of 33 grade 2–3 gliomas (51.5%), 15 of 21 metastases (71.4%) and 1 of 8 other tumours. Both chemotherapy and radiotherapy were documented in 17 grade 4 patients (40.5%) and 9 grade 2–3 patients (27.3%); the dataset records the modalities administered but not the regimen, dose or number of cycles, so completion of the Stupp protocol cannot be verified. Adjuvant stereotactic radiosurgery was recorded in 6 of 21 metastases patients (28.6%). Twenty-five patients (24.0%) had received oncological treatment before surgery, including 15 of 21 metastases patients (71.4%) and 10 glioma patients operated for recurrent, previously treated disease. Treatment was ascertained from free-text clinical records and coded treatment fields, which were incomplete or discordant in a minority of patients, so these proportions represent documented rather than administered treatment.

### 3.3. Genetic Mutations

An explicit IDH result was recorded in 17 of the 42 WHO grade 4 gliomas (14 wild-type, one of them reported as probable wild-type, and 3 mutant). In 22 cases, the status was presumed from the reported diagnosis: 21 recorded as glioblastoma were presumed to be wild-type, and one recorded as WHO grade IV astrocytoma was presumed to be mutant. The wild-type presumption is weaker than it appears. Glioblastoma has been defined as IDH-wildtype only since the fifth edition of the WHO classification, and 17 of the 21 diagnoses were reported with the Roman-numeral grading of the earlier editions, under which glioblastoma was either not defined by IDH status or could be IDH-wildtype, IDH-mutant or not otherwise specified; two further reports stated no grade, and, in three of the 21, IDH was listed in the mutation field without a result. The presumed wild-type group may therefore include IDH-mutant tumours, and survival is also reported for the 14 patients with a recorded wild-type result (Section 3.7). Three grade 4 gliomas could not be classified: two carried no IDH-defining entity, and one labelled glioblastoma had recorded ATRX and TP53 alterations, a profile more consistent with IDH-mutant astrocytoma, so no presumption was made. On this basis, 35 grade 4 gliomas (83.3%) were classified as IDH wild-type (14 recorded, 21 presumed), 4 (9.5%) as IDH-mutant and 3 (7.1%) as undetermined. Among the 33 WHO grade 2–3 gliomas, an explicit result was recorded in 11 and presumed from the reported diagnosis in 19, giving 30 (90.9%) classified as IDH-mutant; the same caveat applies, because before the fifth edition a diffuse or anaplastic astrocytoma could be IDH-wildtype. Of the remaining three, two carried no IDH-defining entity, and one was an anaplastic pleomorphic xanthoastrocytoma, a circumscribed astrocytic glioma that is not defined by IDH status. Diagnoses were taken from the reported histopathology and were not centrally reviewed or re-tested for this study. Other recorded markers included TP53 and ATRX alterations (Table 6). MGMT promoter methylation status and 1p/19q codeletion were not recorded in the dataset and could not be reported.

### 3.4. WHO Grade 4 Glioma

The WHO grade 4 glioma subgroup comprised 42 patients with a mean age of 56.2 years (SD ± 13.6). Tumours were most commonly located in the frontal lobe (42.9%) and parietal lobe (33.3%). Preoperatively, 24/42 patients (57.1%) had motor deficits, 28/42 (66.7%) had headaches or seizures, and 6/42 (14.3%) had visual deficits. Postoperative neurological status was documented in 39 of 42. A motor deficit was present after surgery in 14 of these 39 (35.9%), comprising 10 persistent and 4 new deficits: among the 16 patients without a preoperative motor deficit and with documented status, 4 (25.0%) developed a new deficit, and of the 23 with a preoperative deficit and documented status, 13 improved or resolved and 10 persisted. A visual deficit was present after surgery in 5 of 39 (12.8%), including 2 new deficits among 33 patients at risk. Ten patients (23.8%) had a postoperative complication: a new or worsened neurological deficit in eight, a medical complication (urinary tract infection) in one and a rehabilitation requirement recorded without further detail in one. A further entry in the complication field recorded tumour recurrence; as this is not a postoperative complication, it was excluded from the count. One of the eight patients with a neurological complication also developed pneumonia, hyponatraemia and hydrocephalus. No postoperative haematoma occurred in this subgroup, and none occurred anywhere in the cohort. Extent of resection was documented in 40 of the 42 patients. No residual contrast-enhancing tumour was described in 9 of these 40 (22.5%), comprising supramaximal resection in 3 and gross total resection in 6; the remaining 31 (77.5%) had residual tumour described, classified as near total in 3 and subtotal or partial in 28. Volumetric residual data were not available, so the reason for incomplete resection was inferred from the operative record. Intraoperative neurophysiological monitoring was used in 40 of these 42 patients, and resection was, in most cases, terminated at a functional boundary defined by preoperative mapping (tractography, TMS or functional MRI) or by intraoperative subcortical stimulation rather than at the radiological margin. At censoring, 34/42 patients (81.0%) had died, of whom 6 had undergone repeat surgery. Across the whole cohort there were 21 reoperations (9 grade 4, 9 grade 2–3, 2 metastases and 1 other), and ultrasound was named in the reoperation title in 9 of these; operation titles record adjuncts inconsistently, so this is likely to understate actual use.

### 3.5. WHO Grade 2–3 Glioma

The WHO grade 2–3 glioma subgroup comprised 33 patients with a mean age of 41.6 years (SD ± 12.5). Tumours were most commonly located in the frontal lobe (66.7%). Preoperatively, 8/33 patients (24.2%) had motor deficits, 30/33 (90.9%) had headaches or seizures, and 3/33 (9.1%) had visual deficits. Postoperative neurological status was documented in 28 of 33, the largest number of undocumented patients in any subgroup. A motor deficit was present after surgery in 7 of these 28 (25.0%), comprising 3 persistent and 4 new deficits: among the 21 patients without a preoperative motor deficit and with documented status, 4 (19.0%) developed a new deficit, and of the 7 with a preoperative deficit and documented status, 4 improved or resolved and 3 persisted. A new postoperative visual deficit was present after surgery in 1 of 28 (3.6%); all three preoperative visual deficits improved or resolved. Ten patients (30.3%) had a postoperative complication: a new or worsened neurological deficit, most often dysphasia, in eight, a pseudomeningocoele in one and a delayed wound problem in one. No postoperative haematoma occurred. Extent of resection was documented in 30 of the 33 patients. No residual tumour was described on postoperative imaging in 16 of these 30 (53.3%), comprising supramaximal resection in 3 and gross total resection in 13; the remaining 14 (46.7%) had residual tumour described, classified as subtotal in 13 and partial in one, with no near total resections. Because these tumours are largely non-enhancing, residual tumour in this subgroup refers to T2-weighted or FLAIR signal abnormality as described in the radiology report rather than to contrast enhancement. At censoring, 32 of 33 patients were alive, and one had died.

### 3.6. Metastases

The metastases subgroup comprised 21 patients with a mean age of 62.8 years (SD ± 9.9). Tumours were most commonly located in the occipital lobe (19.0%) and frontal lobe (19.0%). Preoperatively, 14/21 patients (66.7%) had motor deficits, 12/21 (57.1%) had headaches or seizures and 5/21 (23.8%) had visual deficits. Postoperative neurological status was documented in 20 of 21. A motor deficit was present after surgery in 6 of these 20 (30.0%), comprising 5 persistent and 1 new deficit: among the 7 patients without a preoperative motor deficit and with documented status, 1 (14.3%) developed a new deficit, and of the 13 with a preoperative deficit and documented status, 8 improved or resolved and 5 persisted. A visual deficit was present after surgery in 3 of 20, all persistent; no new visual deficit occurred. Six patients (28.6%) had a postoperative complication: a new neurological deficit in four, including one lacunar infarct; a fall on the ward in one; and a wound infection requiring washout with concurrent medical complications in one. No postoperative haematoma occurred. Extent of resection was documented in 19 of the 21 patients. Gross total resection, with no residual contrast-enhancing tumour described, was achieved in 14 of these 19 (73.7%); the remaining 5 (26.3%) had residual tumour described, in each case because the lesion abutted an eloquent cortical or subcortical structure and resection was stopped at the functional boundary. At censoring, 15/21 patients (71.4%) had died.

### 3.7. Survival and Reoperation

**Figure 4:**
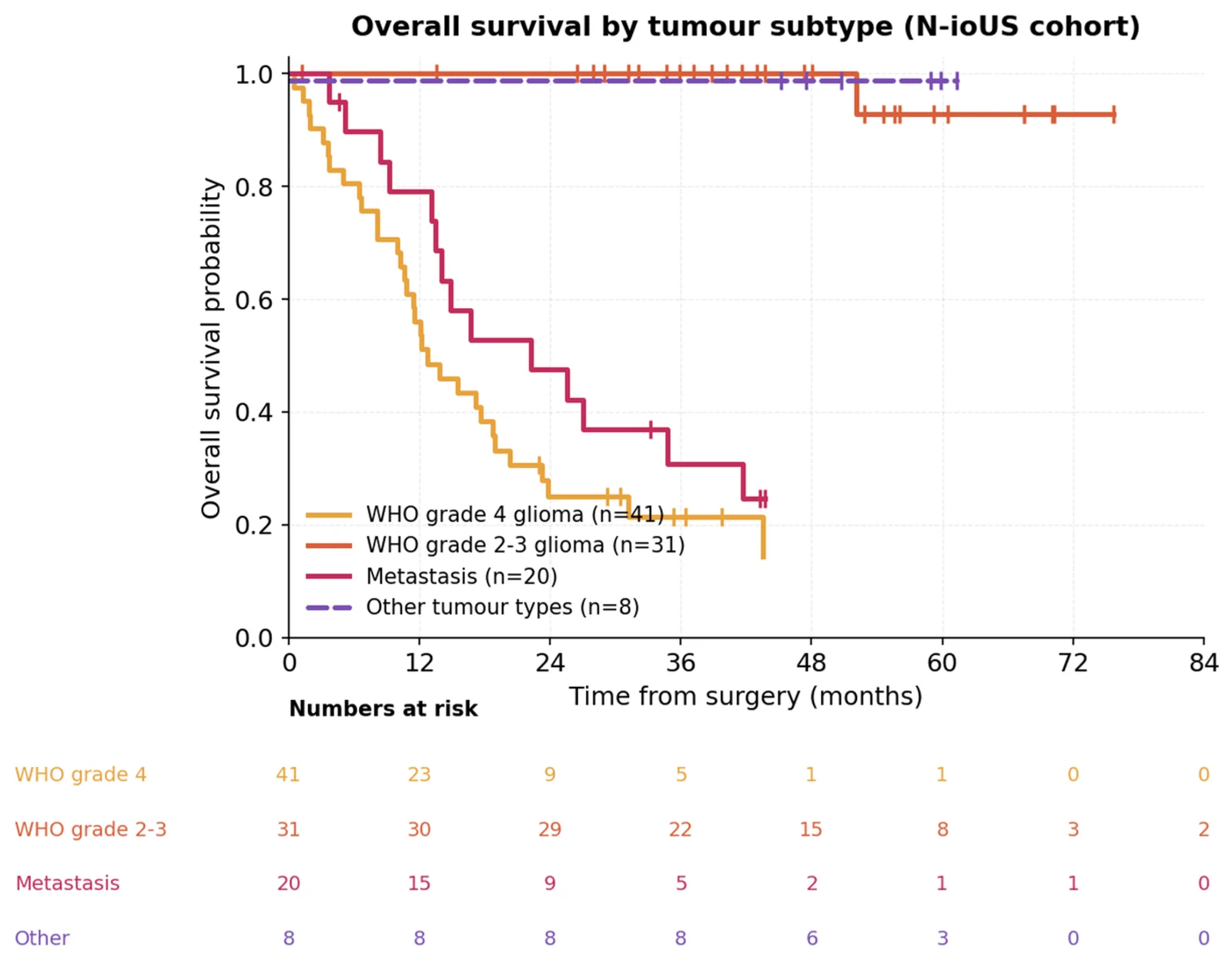
Kaplan–Meier overall survival by tumour subtype (*n* = 100 analysed; grade 4 *n* = 41, grade 2–3 *n* = 31, metastases *n* = 20, other *n* = 8). Vertical ticks denote censored observations, and curves are truncated where fewer than three patients remain at risk. Other tumours recorded no deaths, and WHO grade 2–3 glioma recorded a single death, so both curves remain close to 100%; the “other” curve is drawn dashed and slightly offset below grade 2–3 for visibility.

The survival analysis included 100 of 104 patients. Four patients were excluded for a missing or invalid survival time (one grade 4, two grade 2–3 and one metastases). Overall survival by tumour subtype is shown in Figure 4. In the WHO grade 4 subgroup (41 patients analysed), median follow-up was 36.4 months and 34 patients had died; Kaplan–Meier overall survival was 56.1% at 12 months and 25.1% at 24 months, and median overall survival was 12.7 months. Because IDH status is prognostic in WHO grade 4 glioma, survival was re-estimated descriptively, without formal comparison, for the 34 analysed patients classified as IDH wild-type (recorded or presumed): median overall survival was 12.1 months, with survival of 52.9% at 12 months and 21.1% at 24 months (Table 7). Because most of these classifications were presumed from the reported diagnosis rather than recorded (Section 3.3), survival was also estimated for the 14 patients with a recorded IDH wild-type result: 13 had died, median overall survival was 17.1 months, and survival was 64.3% at 12 months and 14.3% at 24 months. Both estimates rest on small numbers and were not compared formally. Four grade 4 patients were IDH-mutant; three died at 12.7, 31.2 and 62.7 months, and one was censored alive at 30.4 months, so no estimate was made for this group. In the WHO grade 2–3 subgroup (31 patients analysed), median follow-up was 47.3 months with a single death, so median overall survival was not reached. In the metastases subgroup (20 patients analysed), median follow-up was 43.8 months and 15 patients had died; median overall survival was 22.2 months, with overall survival of 79.2% at 12 months and 47.5% at 24 months. Using reoperation for suspected operative-site recurrence as the failure event, freedom from reoperation for suspected local recurrence was 100% at 1 year and 90.9% at 2 years; this endpoint does not capture recurrences managed without surgery and is not a measure of local control. No deaths occurred in the other-tumour subgroup (8 patients; median follow-up 59.0 months).

In an exploratory analysis within the WHO grade 4 subgroup, overall survival did not differ significantly between patients in whom no residual contrast-enhancing tumour was described, comprising supramaximal and gross total resection (*n* = 9), and those in whom residual tumour was described, comprising near total, subtotal and partial resection (*n* = 30) (log-rank *p* = 0.593; Table 7); median overall survival was 15.5 and 12.2 months, respectively. Two patients in whom the extent of resection was not documented were excluded from this comparison. The analysis should be read against the way the extent of resection was determined: category was assigned from the operative note and the postoperative radiology report rather than from volumetric segmentation, so the residual group spans a wide and unquantified range of residual disease, from a few millimetres of margin to substantial residual tumour. With nine patients in the complete-resection group, the comparison had little power to detect a difference, and a volumetric analysis would be required to test whether survival varies within the residual range.

## 4. Discussion

This single-centre retrospective series describes the use of N-ioUS in the resection of functionally eloquent intracranial tumours within a contemporary multimodal neuro-oncology workflow. In 104 patients, N-ioUS was used alongside conventional neuronavigation in 90.4%, 5-ALA in 74.0% and intraoperative neurophysiological monitoring in 94.2%. No residual tumour was described in 22.5% of WHO grade 4 gliomas, 53.3% of WHO grade 2–3 gliomas and 73.7% of metastases, among patients in whom extent of resection was documented. Among patients at risk with documented postoperative status, a new motor deficit occurred in 25.0% of grade 4, 19.0% of grade 2–3 and 14.3% of metastases patients, and 92.9% of patients with preoperative symptoms and documented postoperative status had resolution after surgery. Median overall survival was 12.7 months in WHO grade 4 glioma and 22.2 months in metastases and was not reached in WHO grade 2–3 glioma at a median follow-up of 47.3 months. Because N-ioUS was used in every patient, these figures describe outcomes within the workflow as a whole and cannot be attributed to, or withheld from, N-ioUS.

In WHO grade 4 glioma, median overall survival was 12.7 months at a median follow-up of 36.4 months. For context, the same-institution 5-ALA glioblastoma cohort reported a median overall survival of 17.5 months in the 5-ALA group and 10.6 months in the non-5-ALA group [41]. The present value lies within this range, but the juxtaposition is contextual rather than comparative. Every patient in the present series received N-ioUS; nearly all grade 4 patients also received 5-ALA, and no statistical comparison was made. The two series also overlap in the recruitment period: up to six of the 42 grade 4 patients reported here may appear in the earlier cohort, although the other 36 (85.7%) were operated after it closed. In WHO grade 2–3 glioma, only one death occurred at a median follow-up of 47.3 months, in keeping with the same-institution grade III cohort in which 5-ALA did not confer a survival benefit except where gross total resection was achieved [31]. In metastases, median overall survival was 22.2 months, and freedom from reoperation for suspected local recurrence was 100% at 1 year and 90.9% at 2 years; this endpoint does not capture recurrences managed without surgery and is not comparable to imaging-defined local control, for which published 1-year rates after resection and stereotactic radiosurgery are 70 to 90% [42].

In WHO grade 4 glioma, no residual contrast-enhancing tumour was described in only 22.5% of patients with a documented extent of resection, and overall survival did not differ by extent of resection in an exploratory analysis (no residual versus residual tumour described, log-rank *p* = 0.593, with median overall survival of 15.5 and 12.2 months; Table 7). With only nine patients in the complete-resection group, this comparison had little power, and the absence of a statistically significant difference should not be read as evidence that the extent of resection is unimportant; the finding is at most consistent with the onco-functional nature of modern glioblastoma surgery. Fluorescence-guided resection with 5-ALA extends cytoreduction beyond the contrast-enhancing margin seen on imaging, identifying infiltrative tumours that structural imaging and ultrasound do not depict [36,38,40]. However, in eloquent tumours, the endpoint of resection is set not by residual tumour but by functional boundaries identified through intraoperative neurophysiological monitoring and subcortical stimulation, which stop the resection irrespective of whether the tumour remains [21,22]. Given the infiltrative biology of glioblastoma, in which microscopic extension almost invariably exceeds imaging-defined margins, resection is often terminated at the functional rather than the anatomical border [43]. Because 40 of 42 grade 4 patients also received 5-ALA, this cohort cannot separate the contributions of N-ioUS and 5-ALA, and no independent or comparative treatment effect can be inferred. A new postoperative motor deficit occurred in 4 of 16 grade 4 patients at risk (25.0%), and 10 of 23 preexisting deficits persisted. Neither figure should be read as a low rate. Series confined to tumours involving or abutting eloquent white matter report new postoperative motor deficits in a substantially higher proportion than unselected series, and the lesion-to-tract and cavity-to-tract distances that define this cohort are themselves the strongest predictors of that risk [23,24,25]. The figure is therefore best interpreted as consistent with function-guided resection in a deliberately high-risk population, and it carries no information about any advantage or disadvantage attributable to N-ioUS. These analyses are exploratory and are limited by the small size of some subgroups and by the nine patients whose postoperative status was undocumented.

A similar pattern was seen in WHO grade 2–3 gliomas, where no residual tumour was described in 53.3%. These diffuse tumours infiltrate eloquent white matter despite circumscribed imaging appearances, and the younger age of this subgroup raises the long-term cost of functional injury. Resection was therefore intentionally limited at functional boundaries defined by mapping and tractography; a new motor deficit occurred in 4 of 21 patients at risk (19.0%), most often accompanied by dysphasia in this predominantly left frontal group, and 4 of the 7 preexisting deficits with documented follow-up improved or resolved [18,19,20,21]. Postoperative status was undocumented in 5 of the 33 patients in this subgroup, so these proportions carry the widest uncertainty in the series. 5-ALA was given in 26 of the 33 patients, an off-label use directed at the intraoperative identification of foci of anaplastic transformation within radiologically lower-grade tumours, particularly where heterogeneous restriction of diffusion suggested a higher-grade component [27,28,29,30], rather than at extending the resection of a confirmed grade 2–3 glioma. Fluorescence is inconsistently present in these tumours: in a recent series of 112 grade 2 and grade 3 adult-type diffuse gliomas, 5-ALA fluorescence was seen in 36.6% of cases and sodium fluorescein fluorescence in 16%, and fluorescence was associated with shorter overall and progression-free survival rather than with a larger resection [44]. The argument made below, that fluorescence and ultrasound occupy overlapping decision space, therefore applies with less force in this subgroup than in glioblastoma. The higher rate of complete resection in the lower-grade subgroup, although these tumours are no less infiltrative, reflects how the risk calculation differs between the two groups rather than any difference in imaging. WHO grade 4 gliomas in this series were more often deep, periventricular or closely related to perforating vessels, where extending the resection carries a real risk of ischaemic injury as well as direct tract damage. When median survival is around a year, a new hemiparesis consumes a large fraction of the remaining life, and there is little time to recover from it, so the functional cost of a more aggressive resection is weighted heavily against an uncertain oncological gain. In WHO grade 2–3 gliomas, survival is measured in years, and deficits have time to recover, which justifies pushing the resection further under mapping and monitoring. This asymmetry is recognised in the RANO resect framework, which grades resection separately for contrast-enhancing and non-contrast-enhancing disease [33,34].

In metastases, no residual tumour was described in 73.7%, reflecting the more circumscribed tumour–brain interface. Metastases displace rather than infiltrate the surrounding parenchyma and are usually separated from it by a gliotic pseudocapsule, which gives a large acoustic impedance mismatch and a sharply defined margin on ultrasound. This is the situation in which N-ioUS is expected to be most informative, because the resection endpoint is an anatomical plane that ultrasound can resolve, although this series cannot test that expectation. Its contribution may be smaller in infiltrative glioma, where the endpoint is functional rather than anatomical and no imaging modality defines it, although this series cannot test either expectation. Overall survival is a limited surgical endpoint in this group because prognosis is driven by systemic disease, so local control would be more informative [45]. The rate of reoperation for suspected local recurrence cannot be compared with published local control after resection and stereotactic radiosurgery, and it is not presented as evidence of local control. Adjuvant radiosurgery may reduce the impact of minimal residual disease, lessening the importance of purely anatomical gross total resection [42]. This interpretation is constrained by the surrogate used, as the endpoint was freedom from reoperation for suspected local recurrence rather than imaging-defined local control, and recurrences managed without surgery were not captured.

Brain deformation may occur in several forms, including elastic deformation, where tissue temporarily changes shape before returning to its original configuration; plastic deformation, involving permanent structural alteration following extensive resection or manipulation; and viscoelastic deformation, whereby tissue exhibits time-dependent mechanical responses during prolonged procedures [46,47,48]. Collectively, these phenomena compromise the accuracy of preoperative neuronavigation as surgery progresses and create a need for real-time intraoperative imaging capable of continuously updating anatomical information [5,6,7].

Taken together, these observations suggest a hypothesis, which the present single-arm series cannot test, for why an oncological or functional gain from N-ioUS may be difficult to demonstrate in functionally eloquent tumours. Brain shift and brain deformation are real, and N-ioUS addresses them directly by restoring an accurate anatomical picture during resection. But in functionally eloquent tumours, the anatomical picture is not what limits the operation. Once resection is guided by cortical and subcortical mapping, by intraoperative neurophysiological monitoring and by preoperative tractography, the endpoint is a functional boundary, and a more precise depiction of the tumour margin would not, of itself, be expected to move that boundary. The same applies to the supramaximal resection concept, in which the intended margin lies beyond any imaging-defined edge and so cannot be defined by ultrasound. The effect is compounded by the parallel use of 5-ALA in 74.0% of this cohort and in 40 of 42 WHO grade 4 patients, which independently extends resection beyond the contrast-enhancing margin and therefore potentially occupies much of the same decision space that N-ioUS would otherwise inform. Ultrasound interpretation is operator-dependent, and in a high-volume unit in which surgeons already have extensive experience of resection under mapping guidance, the incremental orientation that N-ioUS provides might be smaller than it would be in a less experienced setting; the advanced modes illustrated in Figure 2 and Figure 3 were used in a minority of cases (strain elastography was documented in 5 index operations and a tubular retractor in 9; Table 5) and represent selected cases rather than routine practice. Whether N-ioUS changed intraoperative conduct is a natural question, and the reasons this series cannot answer it are set out in the Limitations. Prospective studies with a concurrent comparison group, in which the contribution of each intraoperative acquisition to the surgical decision is recorded as it is made, are needed to define the role of N-ioUS in this population.

### Limitations and Strengths

Several limitations apply. The study was retrospective, single-arm, unmatched and non-randomised, and spanned a long period (2011 to 2023) with attendant variability in documentation. Because the use of N-ioUS was an inclusion criterion, every patient received the intervention of interest, and there is no contrast against which its effect could be judged; the observed outcomes describe the multimodal workflow as a whole and cannot be attributed to N-ioUS. Over the same period, eloquent tumours were also operated on at our institution without N-ioUS, but their number cannot be determined reliably, because departmental records from the earlier part of a study period extending back some 15 years are incomplete and were not designed to capture the use or non-use of individual intraoperative adjuncts. An internal comparison was not attempted for two reasons. First, the decision to use N-ioUS was at the discretion of the operating surgeon and was not recorded in a structured field, so patients operated on without it could not be identified systematically by the keyword search used to assemble this cohort, and their number and characteristics are not reliably known. Second, the indication for N-ioUS is likely to have tracked tumour depth, cystic component, proximity to ventricle and surgeon preference, so that even a propensity-matched comparison on histology, age, location and 5-ALA use would leave indication bias unaddressed. The same-institution 5-ALA cohorts cited for context [31,41] recruited between January 2013 and January 2019; six WHO grade 4 and six WHO grade 3 patients in the present series were operated on within that window. Both publications included patients operated on with and without 5-ALA, so any of these twelve may appear in them; the overlap has not been confirmed by record linkage, and twelve is therefore the maximum possible number of shared patients. The remaining 36 of the 42 WHO grade 4 patients (85.7%) and 9 of the 15 WHO grade 3 patients (60.0%) were operated on after January 2019 and cannot have been included. Documentation of the navigation modality was incomplete: the field was blank in eight cases and recorded a non-navigation adjunct in a further two, so the 90.4% figure represents the proportion in which the navigation system used was explicitly recorded rather than a confirmed absence of navigation in the remaining ten cases. Postoperative neurological status was undocumented in 9 of the 104 patients, concentrated in the WHO grade 2–3 subgroup; these patients were excluded from the postoperative denominators, and the reported rates of new and persistent deficit are therefore estimates among documented patients. Deficits were recorded as present or absent rather than graded, so worsening of a preexisting deficit could not be separated from its persistence, and new deficits could not be classified as temporary or permanent because the postoperative entry carries no time point and the timing of recovery was not recorded. The ultrasound acquisition protocol was not prospectively standardised, the number and timing of acquisitions were not recorded, and use of the individual ultrasound modes was ascertained from operation titles that record adjuncts inconsistently; the quantitative performance of N-ioUS in this cohort, including registration accuracy, the magnitude of brain shift compensated, image update latency and any correlation between elastographic strain and histological boundary, was not measured and cannot be derived retrospectively. A change in surgical strategy attributable to the imaging is an outcome measure often reported for intraoperative imaging technologies, including ioUS, intraoperative MRI and intraoperative CT. In this series, however, ioUS was integrated into the surgical workflow at multiple points from dural and cortical exposure onwards, and not only at the end of the resection, which precluded the use of this metric: a baseline acquisition on the intact brain and repeated acquisitions during resection inform orientation continuously, and no single acquisition can be identified as the one that changed the plan. This is a fundamental difference from intraoperative MRI and CT, where the preparation required for image acquisition interrupts the surgical flow to such an extent that their use is largely confined to the end of the resection to assess potentially resectable residual tumour, and where a change of strategy after that single acquisition is therefore a meaningful count. In our experience, an ioUS acquisition made only at the end of resection is also the most difficult to interpret because of artefacts from surgical products, oedema from manipulation, and the mixture of blood and haemostatic material in the cavity. The cohort was heterogeneous, which reflects real-world practice but limits direct comparison between subgroups. Although the survival analysis is based on mature follow-up (median 36 to 59 months across subgroups), it remains descriptive rather than confirmatory because the cohort is heterogeneous, the subgroups are small, and N-ioUS use is confounded with concurrent 5-ALA use; the grade 2–3 and other subgroups had few or no events, so their survival could not be meaningfully estimated. The metastases endpoint of freedom from reoperation for suspected local recurrence does not capture recurrences managed without surgery and is not a measure of local control; an imaging-based operative-site recurrence review would be required to address this limitation. Several further limitations follow from the dataset. The extent of resection was assigned from the operative note and the postoperative radiology report rather than from volumetric segmentation of pre- and postoperative MRI, so the volume-based RANO resect classes could not be applied, and the residual volume within the residual group is unquantified. The extent of resection was not documented in eight of the 104 patients; these are reported separately and were excluded from the resection comparisons. An explicit IDH result was recorded in 28 of the 75 patients with glioma. Status in the remainder was presumed from the reported diagnosis, most of which was issued before the fifth edition of the WHO classification made IDH-wildtype status part of the definition of glioblastoma, so the presumed IDH wild-type grade 4 group may include IDH-mutant tumours. There was no central pathology review, and one grade 4 case carried a diagnosis (anaplastic oligodendroglioma, CNS WHO grade 4) that does not exist in the current classification. MGMT promoter methylation status and 1p/19q codeletion were not recorded, which limits prognostic interpretation of the glioma survival estimates. Progression-free survival could not be derived because serial imaging dates were not captured, which is a particular limitation for the WHO grade 2–3 subgroup, where overall survival is an insensitive endpoint. Cause of death was not available, so deaths could not be attributed to tumour progression rather than to other causes. The strengths of the series are that it describes navigated intraoperative ultrasound within a modern multimodal workflow, that it reflects unselected real-world practice across a broad range of pathologies with mature follow-up, and that it reports functional outcome with explicit denominators in a population defined a priori by proximity to eloquent tracts and cortex.

## 5. Conclusions

In this descriptive single-arm series of functionally eloquent tumours operated within a modern neuro-oncology workflow using fluorescence guidance and preoperative and intraoperative mapping and monitoring, the resection endpoint was set by functional rather than anatomical boundaries, and the rates of complete resection and of new neurological deficit were in keeping with that constraint. Because N-ioUS was used in every patient, the series cannot determine whether N-ioUS improved or failed to improve functional or oncological outcomes; the same-institution cohorts cited here provide context, not a comparison. N-ioUS remains a low-cost and widely accessible adjunct for intraoperative orientation and confirmation of residual tumour, and its contribution may be greatest where the resection endpoint is anatomical, as in metastases, rather than functional. Prospective controlled studies that record intraoperative decision-making as an endpoint are required to define its place within the surgical workflow in differing neuro-oncology patient populations.

## Figures and Tables

**Table 1:** Baseline demographics and tumour characteristics of the whole cohort.

Variable	Number	Percentage (%)
Total patients	104	100
Male	60	57.7
Age, years (mean ± SD)	51.8 ± 15.5	-
Histology		
WHO grade 4 glioma	42	40.4
WHO grade 2–3 glioma	33	31.7
Metastases	21	20.2
Other (ependymoma, haemangioblastoma or grade 1 glioma)	8	7.7
Location *		
Temporal	25	24.0
Frontal	45	43.3
Parietal	26	25.0
Occipital	7	6.7
Posterior fossa	7	6.7
Cerebellar	1	1.0
Other (cingulate gyrus, pterional)	3	2.9
Laterality (approach)		
Left	50	48.1
Right	48	46.2
Bilateral or midline	6	5.8

* Lesions involved more than one lobe in some patients, so location counts exceed the number of patients.

**Table 2:** Neurological status before and after surgery, whole cohort.

Variable	Number	Percentage (%)
Total patients	104	100
Before surgery (*n* = 104)		
Motor deficit	49	47.1
Headache, dizziness, collapse or seizures	78	75.0
Visual deficit	16	15.4
After surgery, documented status (*n* = 95) *		
Motor deficit	28	29.5
Headache, dizziness, collapse or seizures	6	6.3
Visual deficit	10	10.5
Postoperative status undocumented	9	8.7
Change in status ^†^		
New motor deficit (of 49 at risk)	10	20.4
Persistent preexisting motor deficit (of 46)	18	39.1
Improved or resolved motor deficit (of 46)	28	60.9
New visual deficit (of 79 at risk)	3	3.8
Resolution of preoperative symptoms (of 70)	65	92.9

* Postoperative neurological status was undocumented in nine patients, who were excluded from the postoperative denominators rather than assumed to be free of deficit. ^†^ Patients at risk of a new deficit are those without that deficit before surgery and with documented postoperative status. Deficits were recorded as present or absent, so worsening of a preexisting deficit cannot be distinguished from persistence.

**Table 3:** Surgical adjuncts and extent of resection by tumour subtype.

Variable	Total	Grade 4 (*n*)	Grade 2–3 (*n*)	Metastases (*n*)	Other (*n*)
Total patients	104	42	33	21	8
Surgical adjuncts and mapping *					
Navigated intraoperative ultrasound	104	42	33	21	8
Optical neuronavigation (Medtronic)	82	32	28	18	4
Electromagnetic neuronavigation (AxiEM)	12	5	3	2	2
Intraoperative neurophysiological monitoring	98	40	32	19	7
Tractography	38	11	18	6	3
Transcranial magnetic stimulation	23	7	14	1	1
Functional MRI	7	0	6	0	1
5-ALA fluorescence	77	40	26	8	3
Indocyanine green fluorescence	13	7	5	1	0
Augmented reality	10	4	4	2	0
Extent of resection ^†^					
Supramaximal resection	6	3	3	0	0
Gross total resection	37	6	13	14	4
Near total resection	3	3	0	0	0
Subtotal or partial resection	50	28	14	5	3
Extent of resection not documented	8	2	3	2	1
No residual tumour described (supramaximal or gross total), *n*/documented	43/96	9/40	16/30	14/19	4/7
No residual tumour described, % of documented	44.8	22.5	53.3	73.7	57.1

* Navigated intraoperative ultrasound was used in all 104 patients, which was an inclusion criterion. Conventional neuronavigation was used in addition in 94 of 104 patients (90.4%). More than one adjunct was used in most patients, so these rows are not mutually exclusive and do not sum to the cohort total. ^†^ Extent of resection was assigned from the operative note and the postoperative radiology report; the categories are mutually exclusive. Volumetric segmentation was not available, so the volume-based RANO resect classes were not applied. Where the operative note and the radiology report disagreed, or the entry was qualified as uncertain, the lower category was taken. For contrast-enhancing tumours, no residual tumour refers to a contrast-enhancing tumour; for largely non-enhancing WHO grade 2–3 gliomas, it refers to residual tumour on T2-weighted or FLAIR imaging as described in the radiology report. Percentages in the final row use the number of patients in whom extent of resection was documented as the denominator (96 of 104 overall; 40 grade 4, 30 grade 2–3, 19 metastases and 7 other).

**Table 4:** Neurological status, complications, length of stay and oncological treatment by tumour subtype.

Variable	Total	Grade 4 (*n*)	Grade 2–3 (*n*)	Metastases (*n*)	Other (*n*)
Total patients	104	42	33	21	8
Length of stay, days (mean ± SD) *	9.6 ± 11.5	9.6 ± 10.8	6.6 ± 3.1	12.1 ± 15.7	13.5 ± 19.0
Alive at censoring	54	8	32	6	8
Neurological status before surgery					
Motor deficit	49	24	8	14	3
Headache, dizziness, collapse or seizures	78	28	30	12	8
Visual deficit	16	6	3	5	2
Neurological status after surgery ^†^					
Postoperative status documented	95	39	28	20	8
Motor deficit	28	14	7	6	1
Headache, dizziness, collapse or seizures	6	2	2	0	2
Visual deficit	10	5	1	3	1
Change in motor status ^‡^					
New motor deficit, *n*/at risk	10/49	4/16	4/21	1/7	1/5
New motor deficit, % at risk	20.4	25.0	19.0	14.3	20.0
Persistent preexisting motor deficit, *n*/documented	18/46	10/23	3/7	5/13	0/3
Improved or resolved motor deficit, *n*/documented	28/46	13/23	4/7	8/13	3/3
New visual deficit, *n*/at risk	3/79	2/33	1/25	0/15	0/6
Complications					
Any postoperative complication	29	10	10	6	3
Postoperative haematoma	0	0	0	0	0
Oncological treatment ^§^					
Any documented postoperative treatment	68	35	17	15	1
Chemotherapy and radiotherapy both documented	31	17	9	4	1
Chemotherapy only	23	14	6	3	0
Radiotherapy only	14	4	2	8	0
Adjuvant stereotactic radiosurgery	6	0	0	6	0
No postoperative treatment documented	36	7	16	6	7
Any documented preoperative treatment	25	6	4	15	0

* Length of stay was recorded in 100 of 104 patients. ^†^ Postoperative neurological status was undocumented in 9 patients (3 grade 4, 5 grade 2–3 and 1 metastases), who are excluded from the postoperative rows rather than assumed to be free of deficit. ^‡^ Patients at risk of a new deficit are those without that deficit before surgery and with documented postoperative status. Deficits were recorded as present or absent, so worsening of a preexisting deficit cannot be distinguished from persistence. ^§^ Treatment was ascertained from free-text clinical records and coded treatment fields. Both modalities were counted when the free text named chemotherapy and radiotherapy or the coded chemoradiotherapy field was set; a single coded field contradicting the free text was not taken as corroboration. The dataset records the modalities administered but not the regimen, dose or number of cycles, so completion of the Stupp protocol cannot be verified. Adjuvant stereotactic radiosurgery is a subset of the radiotherapy-containing rows and is not additive. These proportions represent documented rather than administered treatment.

**Table 5:** Documented use of navigated intraoperative ultrasound modalities and related adjuncts by tumour subtype.

Variable	Total	Grade 4 (*n*)	Grade 2–3 (*n*)	Metastases (*n*)	Other (*n*)
Total patients	104	42	33	21	8
Navigated intraoperative ultrasound (inclusion criterion)	104	42	33	21	8
Ultrasound named in the operation title	69	29	20	13	7
Fusion with navigated MRI named explicitly	19	10	5	3	1
Strain elastography, index operation	5	2	1	2	0
Strain elastography, reoperation only	2	1	1	0	0
Contrast-enhanced ultrasound	0	0	0	0	0
Tubular retractor or transsulcal parafascicular approach	9	7	0	1	1
Endoscope-assisted	5	3	0	1	1
Awake or asleep-awake-asleep procedure	36	13	21	2	0
Reoperation during follow-up	21	9	9	2	1
Ultrasound named in the reoperation title	9	4	4	1	0

Counts were derived from the operation title of the index procedure and of any reoperation and from the structured adjunct fields of the departmental database. Operation titles record adjuncts inconsistently, so all rows other than the first are likely to understate actual use. Procedures were performed under 9 consultant neurosurgeons, four of whom accounted for 96 of the 104 operations. Whether ultrasound altered intraoperative conduct was not recorded in a structured field.

**Table 6:** Demographics and molecular findings by tumour subtype.

Variable	Total	Grade 4 (*n*)	Grade 2–3 (*n*)	Metastases (*n*)	Other (*n*)
Total patients	104	42	33	21	8
Male	60	28	16	11	5
Age, years (mean ± SD)	51.8 ± 15.5	56.2 ± 13.6	41.6 ± 12.5	62.8 ± 9.9	42.3 ± 19.5
IDH status ^¶^					
IDH wild-type (recorded or presumed)	35	35	0	n/a	n/a
Of which an explicit IDH result was recorded	14	14	0	n/a	n/a
IDH-mutant (recorded or presumed)	34	4	30	n/a	n/a
Of which an explicit IDH result was recorded	14	3	11	n/a	n/a
Not determined or not an IDH-defined tumour type	6	3	3	n/a	n/a
Other molecular markers recorded ^#^					
TP53	3	3	0	0	0
ATRX	4	3	1	0	0
TERT promoter	3	3	0	0	0

^¶^ IDH status was taken from an explicit IDH result where one was recorded, and, otherwise, presumed from the reported diagnosis, on the basis that under the fifth edition of the WHO Classification of Tumours of the Central Nervous System, glioblastoma is IDH-wildtype by definition, and adult-type diffuse gliomas of grades 2 or 3 are IDH-mutant astrocytomas or oligodendrogliomas. Most diagnoses were reported under earlier editions, in which these equivalences did not hold, so presumed status is uncertain (Section 3.3). An explicit result was available in 28 of the 75 patients with glioma; the indented rows give these numbers. Cases recorded only as high-grade glioma without a definitive entity, one case labelled glioblastoma whose recorded ATRX and TP53 alterations are more consistent with IDH-mutant astrocytoma, and one anaplastic pleomorphic xanthoastrocytoma are shown as not determined or not IDH-defined. IDH status does not apply to metastases or to the non-glial tumours in the other subgroup, so the totals in this section refer to the 75 patients with glioma. MGMT promoter methylation status and 1p/19q codeletion were not recorded in the dataset and could not be reported. ^#^ Additional molecular markers were recorded ad hoc in the histopathology reports rather than tested systematically, so these counts reflect what was documented and understate the proportion tested.

**Table 7:** Exploratory overall survival analyses within the WHO grade 4 glioma subgroup.

Analysis	Group	*n*	Median OS, Months	Log-Rank *p*
Extent of resection *	No residual contrast-enhancing tumour described (supramaximal or gross total resection)	9	15.5	0.593
	Residual tumour described (near total, subtotal or partial resection)	30	12.2	
IDH status ^†^	IDH wild-type, recorded or presumed	34	12.1	Not tested
	IDH wild-type, recorded result only	14	17.1	Not tested
	IDH-mutant, recorded or presumed	4	Not estimated ^‡^	Not tested

* Thirty-nine of 41 patients analysed; two patients in whom extent of resection was not documented were excluded. Extent of resection was assigned from the operative note and the postoperative radiology report, not from volumetric segmentation. ^†^ Descriptive only; no statistical comparison by IDH status was made. Thirty-eight of 41 patients analysed; three patients whose IDH status could not be determined were excluded. IDH status was taken from an explicit result where recorded and otherwise presumed from the reported diagnosis (see Section 3.3 and Table 6); the recorded-only row is a subset of the row above it. Overall survival was 52.9% at 12 months and 21.1% at 24 months in the recorded or presumed wild-type group and 64.3% and 14.3% in the recorded-only group. The IDH-mutant group is too small to support inference. ^‡^ Three of the four IDH-mutant patients died at 12.7, 31.2, and 62.7 months and one was censored alive at 30.4 months.

## Data Availability

The data presented in this study are available on reasonable request from the corresponding author. The data are not publicly available because they contain information that could compromise the privacy of research participants.

## Supplementary Materials

The following supporting information can be downloaded at: https://www.mdpi.com/article/10.3390/jpm16100516/s1. File S1: STROBE checklist for cohort studies.
