# Supplementary material for: Navigated Intraoperative Ultrasound in Functionally Eloquent Tumour Resection: A Descriptive Single-Centre Series Within the Modern Multimodal Neuro-Oncology Surgery Workflow
Source: J Pers Med. 2026 Oct 2;16(10):516. doi: 10.3390/jpm16100516 (PMC13705947; doi:10.3390/jpm16100516)
Supplement: Supplementary file 1 [file jpm-16-00516-s001.zip › jpm-4506307-supplementary.pdf]

File S1. STROBE Statement—checklist of items that should be included in reports of cohort studies

Manuscript: *The Role of Navigated Intraoperative Ultrasound (N-ioUS) in Functional-Eloquent Tumour Resection within the Modern Neuro-Oncology Surgery Workflow*. Fayez F. et al. Submitted to the Journal of Personalized Medicine (manuscript jpm-4506307).

Page and line numbers refer to the submitted manuscript file, which carries continuous line numbers in the left margin.

	Item No.	Recommendation	Page No.	Relevant text from manuscript
**Title and abstract**				
Title and abstract	1	(a) Indicate the study's design with a commonly used term in the title or the abstract	1, 2	**p.2, lines 17–20:** "Methods: Retrospective cohort study reported per STROBE of 104 consecutive patients who underwent N-ioUS-guided resection of functional-eloquent intracranial tumours at a tertiary neurosurgical centre between 2011 and 2023."<br>**p.3, lines 97–98:** "In this retrospective cohort study, we present a large single-centre series of patients undergoing intracranial tumour resection using navigated intraoperative ultrasound…"
		(b) Provide in the abstract an informative and balanced summary of what was done and what was found	2	**p.2, lines 13–36:** *Structured abstract with Background/Objectives, Methods, Results and Conclusions.*<br>**p.2, lines 24–32:** "Complete resection of contrast-enhancing tumour (RANO RESECT class 1 or 2) was achieved in 22.5% of grade 4 gliomas, 53.3% of grade 2–3 gliomas and 73.7% of metastases, among the 96 patients with a documented extent of resection… Because nearly all grade 4 patients also received 5-ALA, no independent effect of N-ioUS could be inferred."
**Introduction**				
Background/rationale	2	Explain the scientific background and rationale for the investigation being reported	2, 3	**p.2, lines 49–52:** "One of the most important of these phenomena is brain shift, which occurs secondary to cerebrospinal fluid drainage, tumour debulking, gravity, osmotic changes, and patient positioning, resulting in progressive displacement of intracranial structures throughout surgery."<br>**p.3, lines 56–60:** "Navigated intraoperative ultrasound (N-ioUS), which integrates ultrasound imaging with preoperative MRI or CT-based neuronavigation, enables real-time image fusion and dynamic intraoperative updating of anatomical relationships."<br>**p.3, lines 94–96:** "The extent of resection achievable in this setting is therefore constrained by function rather than by image quality, and any imaging adjunct intended to improve resection must be judged against that constraint."
Objectives	3	State specific objectives, including any prespecified hypotheses	2, 3	**p.2, lines 16–17:** "We evaluated the safety and surgical outcomes of N-ioUS in a heterogeneous single-centre cohort."<br>**p.3, lines 97–100:** "…we present a large single-centre series of patients undergoing intracranial tumour resection using navigated intraoperative ultrasound between 2011 and 2023, where the impact of N-ioUS is considered within a contemporary multimodal neuro-oncology surgical workflow."<br>*No formal statistical hypothesis was prespecified; the survival comparison is declared exploratory (p.4–5, lines 153–157).*
**Methods**				
Study design	4	Present key elements of study design early in the paper	2, 3	**p.3, lines 102–105:** "This study was conducted per the STROBE guidelines for cohort studies. We performed a retrospective cohort analysis encompassing all patients who underwent tumour resections using navigated intraoperative ultrasound (N-ioUS) at our institution between 2011 and 2023."

	Item No.	Recommendation	Page No.	Relevant text from manuscript
				**p.2, lines 17–20:** *Design also stated in the first line of the abstract Methods.*
Setting	5	Describe the setting, locations, and relevant dates, including periods of recruitment, exposure, follow-up, and data collection	3, 4, 14	**p.3–4, lines 103–107:** "…all patients who underwent tumour resections using navigated intraoperative ultrasound (N-ioUS) at our institution between 2011 and 2023. The study included patients of all age groups referred by the neuro-oncology multidisciplinary team (MDT). The indication for the use of N-ioUS was at the discretion of the surgical team responsible for the treatment."<br>**p.4, lines 149–151:** "Overall survival was estimated using the Kaplan-Meier method, measured from the date of surgery to death or, for patients who remained alive, to the date of last follow-up, at which point observations were censored."<br>**p.14, lines 355–371:** *Median follow-up reported for each subgroup (36.4, 47.3, 43.8 and 59.0 months).*
Participants	6	(a) Cohort study—Give the eligibility criteria, and the sources and methods of selection of participants. Describe methods of follow-up	4	**p.4, lines 108–114:** "Only patients with tumours in functional-eloquent locations were included. Eloquence was defined a priori as a lesion-to-tract distance of less than 10 mm from an eloquent white matter tract, most commonly the corticospinal tract or the arcuate fasciculus, on preoperative tractography, or radiological involvement of the basal ganglia, internal capsule, insula, or of cortex with an established motor or language function."<br>**p.4, lines 115–119:** "To identify eligible cases, we systematically screened the electronic patient records at our centre using the keywords "ioUS," "ultrasound," "USS," and "Intraoperative Navigation" within all neuro-oncology notes. Records were then reviewed against the eloquence criteria above. All resection approaches were included in the analysis, with no exclusions based on the method of application of ioUS."<br>**p.4, lines 149–151:** *Follow-up was through routine clinical records to death or last recorded contact, at which point observations were censored.*
		(b) Cohort study—For matched studies, give matching criteria and number of exposed and unexposed	n/a	*Not applicable. This is an unmatched single-arm cohort in which every patient received N-ioUS, stated at p.19, lines 521–522 ("The study was retrospective, unmatched and non-randomised") and p.7, line 199 ("All patients underwent surgery using N-ioUS"). There is no internal unexposed group; comparison is with previously published same-institution cohorts.*
Variables	7	Clearly define all outcomes, exposures, predictors, potential confounders, and effect modifiers. Give diagnostic criteria, if applicable	4, 12	**p.4, lines 120–127:** "Data collection focused on patient demographics (including age at the time of surgery, sex, type of surgery, adjuvant therapy, lesion location and pre-operative neurological function), methods of surgical mapping… and outcome variables such as post-operative neurological function, the number of days to discharge, extent of resection, post-op complications, histological findings and adjuvant therapy."<br>**p.4, lines 128–144:** *Extent of resection defined in full, with the mapping to RANO RESECT classes 1–2 versus 3–4 and the rule applied where sources disagreed.*<br>**p.12, lines 269–283:** *Diagnostic criteria for IDH status under the fifth edition of the WHO classification, including which cases were explicit and which inferred.*
Data sources/ measurement	8*	For each variable of interest, give sources of data and details of methods of assessment (measurement). Describe comparability of assessment methods if there is more than one group	4, 8, 12	**p.4, lines 132–137:** "Volumetric segmentation of pre- and postoperative MRI was not performed, so resection was categorised from the operative note and the postoperative radiology report… Where the two sources disagreed, or the entry was qualified as uncertain, the lower category was taken."<br>**p.8, lines 222–228:** "Treatment was ascertained from free-text clinical records and coded treatment fields. Both modalities were counted when the free text named chemotherapy and radiotherapy or

	Item No.	Recommendation	Page No.	Relevant text from manuscript
				the coded chemoradiotherapy field was set; a single coded field contradicting the free text was not taken as corroboration."<br>**p.12, lines 280–281:** "Diagnoses were taken from the reported histopathology and were not centrally reviewed or re-tested for this study."<br>*The same records, definitions and ascertainment rules were applied to every tumour subtype, so assessment is comparable across groups.*
Bias	9	Describe any efforts to address potential sources of bias	4, 8, 16, 19	**p.4, lines 135–137:** "Where the two sources disagreed, or the entry was qualified as uncertain, the lower category was taken." *Conservative rule against overstating extent of resection.*<br>**p.8, lines 222–228:** *Treatment counted only on corroborated documentation, reported as documented rather than administered treatment.*<br>**p.16, lines 401–406:** "The analysis should be read against the way extent of resection was determined… the residual group spans a wide and unquantified range of residual disease."<br>**p.19, lines 521–538:** *Confounding by concurrent 5-ALA and mapping use, incomplete documentation of navigation modality, and the reoperation surrogate for local control are each identified and their likely direction stated.*
Study size	10	Explain how the study size was arrived at	3, 4	**p.3, lines 103–105:** "…a retrospective cohort analysis encompassing all patients who underwent tumour resections using navigated intraoperative ultrasound (N-ioUS) at our institution between 2011 and 2023."<br>**p.4, lines 115–119:** *Size was determined by the number of consecutive eligible cases identified by keyword screening of the electronic record over the study period. No a priori sample size calculation was performed and none is reported, since the study is descriptive.*
Quantitative variables	11	Explain how quantitative variables were handled in the analyses. If applicable, describe which groupings were chosen and why	4, 16	**p.4, lines 147–149:** "Continuous variables were summarised as means with standard deviations (SD) and categorical variables as frequencies and percentages."<br>**p.4, lines 137–144:** "Because the RANO RESECT classes are defined by residual tumour volume, they could not be applied in their published form; supramaximal and gross total resection were taken to correspond to classes 1 and 2… and near total, subtotal and partial resection to classes 3 and 4. Results are therefore presented as complete resection of contrast-enhancing tumour versus residual tumour."<br>**p.16, lines 395–399:** *The same two-group split is used for the survival comparison. Age was analysed as a continuous variable and was not categorised.*
Statistical methods	12	(a) Describe all statistical methods, including those used to control for confounding	4, 5	**p.4, lines 146–155:** "Statistical analyses were conducted using R (version 4.3.2; The R Foundation for Statistical Computing) and RStudio (version 2023.12.0+369)… Overall survival was estimated using the Kaplan-Meier method… As a single exploratory analysis, overall survival by extent of resection within the WHO grade 4 subgroup was compared using the log-rank test, with statistical significance defined as $p<0.05$."<br>**p.5, lines 156–157:** "…no other inferential testing was performed, and all survival findings are regarded as exploratory and hypothesis-generating."<br>*No multivariable modelling was performed, so no statistical control for confounding was applied. Confounding is addressed in the Discussion rather than in the analysis (p.19, lines 531–536).*
		(b) Describe any methods used to examine subgroups and interactions	4, 5, 14	**p.4, lines 153–155:** "…overall survival by extent of resection within the WHO grade 4 subgroup was compared using the log-rank test."

	Item No.	Recommendation	Page No.	Relevant text from manuscript
				**p.14, lines 355–371:** *Kaplan-Meier estimates presented separately for the four histological subgroups (grade 4, grade 2–3, metastasis, other).*<br>**p.14, lines 358–364:** "Because IDH status is prognostic in WHO grade 4 glioma, survival was re-estimated for the 34 IDH wild-type patients alone."<br>*No formal interaction testing was performed.*
		(c) Explain how missing data were addressed	4, 8, 14, 19	**p.4, lines 151–153:** "Patients with a missing or invalid survival time were excluded from the survival analysis, which therefore included 100 of 104 patients."<br>**p.4, lines 141–144:** "…percentages calculated among the patients in whom extent of resection was documented; patients with no documented extent of resection are reported separately and were excluded from the resection comparison."<br>**p.8, lines 228–229 and p.10, lines 249–250:** *Denominators stated wherever they differ from 104 (for example, length of stay recorded in 100 of 104 patients).*<br>*Analyses are complete-case throughout; no imputation was performed.*
		(d) Cohort study—If applicable, explain how loss to follow-up was addressed	4, 14	**p.4, lines 149–151:** "…measured from the date of surgery to death or, for patients who remained alive, to the date of last follow-up, at which point observations were censored."<br>**p.15, lines 373–375 and p.16, lines 383–384:** *Censored observations are marked by vertical ticks on each Kaplan-Meier curve, and curves are truncated where fewer than three patients remain at risk.*
		(e) Describe any sensitivity analyses	14	**p.14, lines 358–361:** "Because IDH status is prognostic in WHO grade 4 glioma, survival was re-estimated for the 34 IDH wild-type patients alone: median overall survival was 12.1 months, with survival of 52.9% at 12 months and 21.1% at 24 months (Figure 6)."<br>*This restriction to a molecularly homogeneous subgroup is the only sensitivity analysis performed. No other sensitivity analyses were undertaken.*
**Results**				
Participants	13*	(a) Report numbers of individuals at each stage of study—eg numbers potentially eligible, examined for eligibility, confirmed eligible, included in the study, completing follow-up, and analysed	4, 5, 13, 14	**p.4, lines 115–118:** *Case ascertainment by keyword screening of all neuro-oncology electronic records, then review against the eloquence criteria.*<br>**p.5, line 160:** "A total of 104 patients underwent intracranial tumour resection using N-ioUS."<br>**p.14, line 353:** "The survival analysis included 100 of 104 patients."<br>**p.13–14, lines 311–348:** *Numbers with a documented extent of resection given for each subgroup: 40 of 42 grade 4, 30 of 33 grade 2–3 and 19 of 21 metastasis.*<br>*The number of records screened before eligibility review is not recorded and cannot be reported.*
		(b) Give reasons for non-participation at each stage	14, 16, 19	**p.14, lines 353–354:** "Four patients were excluded for a missing or invalid survival time (one grade 4, two grade 2–3 and one metastasis)."<br>**p.16, lines 400–401:** "Two patients in whom extent of resection was not documented were excluded from this comparison."<br>**p.19, lines 547–549:** "Extent of resection was not documented in eight of the 104 patients; these are reported separately and were excluded from the resection comparisons."

	Item No.	Recommendation	Page No.	Relevant text from manuscript
		(c) Consider use of a flow diagram	n/a	*A flow diagram is not included. All stage-by-stage numbers are reported in the text at p.5, line 160, p.14, lines 353–354 and p.19, lines 547–549. A diagram would add little for a single-arm cohort with two documented exclusion reasons.*
Descriptive data	14*	(a) Give characteristics of study participants (eg demographic, clinical, social) and information on exposures and potential confounders	5, 6, 7, 12	**p.5, lines 160–171:** "A total of 104 patients underwent intracranial tumour resection using N-ioUS. The cohort was predominantly male (57.7%) with a mean age of 51.8 years (SD ± 15.5). The most common histological subtype was WHO grade 4 glioma (40.4%)…"<br>**p.5, line 172:** *Table 1, baseline demographics and tumour characteristics of the whole cohort.*<br>**p.6, lines 181–190 and Table 2:** *Preoperative and postoperative neurological deficits.*<br>**p.7, lines 199–216 and Tables 3 and 4:** *Co-interventions, which are the principal potential confounders: neuronavigation 90.4%, IONM 94.2%, 5-ALA 74.0%, tractography 36.5%, TMS 22.1%, fMRI 6.7%.*<br>**p.12, line 284:** *Table 5, demographics and molecular findings by tumour subtype.*
		(b) Indicate number of participants with missing data for each variable of interest	6, 8, 10, 12, 19	**p.6, lines 192–193:** "The mean length of hospital stay across the cohort was 9.6 days (SD ± 11.5), recorded in 100 of 104 patients."<br>**p.8–9, lines 229–237:** *Extent of resection documented in 96 of 104 overall (40 grade 4, 30 grade 2–3, 19 metastasis, 7 other).*<br>**p.12, lines 282–283:** "MGMT promoter methylation status and 1p/19q codeletion were not recorded in the dataset and could not be reported."<br>**p.19, lines 524–528:** "Documentation of the navigation modality was incomplete: the field was blank in eight cases and recorded a non-navigation adjunct in a further two."<br>**p.19, lines 549–551:** *Explicit IDH result available in 28 of the 75 patients with glioma.*
		(c) Cohort study—Summarise follow-up time (eg, average and total amount)	14, 19	**p.14, lines 355–371:** "In the WHO grade 4 subgroup (41 patients analysed), median follow-up was 36.4 months… In the WHO grade 2–3 subgroup (31 patients analysed), median follow-up was 47.3 months… In the metastasis subgroup (20 patients analysed), median follow-up was 43.8 months… the other-tumour subgroup (8 patients; median follow-up 59.0 months)."<br>**p.19, lines 531–533:** "…the survival analysis is now based on mature follow-up (median 36 to 59 months across subgroups)."
Outcome data	15*	Cohort study—Report numbers of outcome events or summary measures over time	6, 13, 14	**p.6, lines 186–189:** "Of the 49 patients with a preoperative motor deficit, 31 (63.3%) improved or resolved, and 10 patients developed a new motor deficit."<br>**p.13, lines 311–315:** "Extent of resection was documented in 40 of the 42 patients. Complete resection of the contrast-enhancing tumour was achieved in 9 of these 40 (22.5%)… the remaining 31 (77.5%) had residual tumour."<br>**p.13–14, lines 320, 337–338, 350–351:** *Deaths at censoring: 34 of 42 grade 4 (81.0%), 1 of 33 grade 2–3, 15 of 21 metastasis (71.4%).*<br>**p.14, lines 355–370:** *Kaplan-Meier survival at 12 and 24 months and median overall survival for each subgroup, and freedom from reoperation for suspected local recurrence of 100% at 1 year and 90.9% at 2 years.*
Main results	16	(a) Give unadjusted estimates and, if applicable, confounder-adjusted estimates and their precision (eg, 95% confidence	14, 16, 19	**p.14, lines 355–358:** "Kaplan-Meier overall survival was 56.1% at 12 months and 25.1% at 24 months, and median overall survival was 12.7 months."

	Item No.	Recommendation	Page No.	Relevant text from manuscript
		interval). Make clear which confounders were adjusted for and why they were included		**p.16, lines 395–400:** "…overall survival did not differ significantly between patients who underwent complete resection of the contrast-enhancing tumour… and those left with residual tumour… (log-rank p = 0.593; Figure 5); median overall survival was 15.5 and 12.2 months respectively."<br>*All estimates are unadjusted. No confounder-adjusted estimates are presented because no multivariable modelling was performed; the reason is given at p.5, lines 155–157 and p.19, lines 531–536. Confidence intervals around the Kaplan-Meier estimates are not reported, which is a reporting limitation of this descriptive analysis.*
		(b) Report category boundaries when continuous variables were categorized	4, 16	**p.4, lines 128–144:** *Boundaries of the extent-of-resection categories and their mapping to RANO RESECT classes 1–2 versus 3–4 given in full.*<br>**p.4, lines 109–112:** "Eloquence was defined a priori as a lesion-to-tract distance of less than 10 mm from an eloquent white matter tract…"<br>*Age and length of stay were analysed as continuous variables and were not categorised.*
		(c) If relevant, consider translating estimates of relative risk into absolute risk for a meaningful time period	2, 14	*No relative risk, hazard ratio or odds ratio is reported, so translation does not arise. Results are already given on the absolute scale.*<br>**p.14, lines 356–368:** "Kaplan-Meier overall survival was 56.1% at 12 months and 25.1% at 24 months… median overall survival was 22.2 months, with overall survival of 79.2% at 12 months and 47.5% at 24 months."
Other analyses	17	Report other analyses done—eg analyses of subgroups and interactions, and sensitivity analyses	14, 16, 17	**p.14, lines 358–364:** *Survival re-estimated in IDH wild-type patients alone, and the IDH-mutant comparison reported as uninformative (log-rank p = 0.087).*<br>**p.14, lines 368–370:** "Using reoperation for suspected operative-site recurrence as the failure event, freedom from reoperation for suspected local recurrence was 100% at 1 year and 90.9% at 2 years."<br>**p.16, lines 395–406:** *Exploratory extent-of-resection survival comparison in the WHO grade 4 subgroup, with its interpretation constrained by the non-volumetric definition of residual disease.*<br>**p.17, lines 420–431:** *Descriptive comparison with two previously published same-institution 5-ALA cohorts.*
**Discussion**				
Key results	18	Summarise key results with reference to study objectives	16, 17	**p.16–17, lines 408–419:** "This single-centre retrospective series evaluated N-ioUS in the resection of functional-eloquent intracranial tumours within a contemporary multimodal neuro-oncology workflow. In 104 patients, N-ioUS was used alongside conventional neuronavigation in 90.4%, 5-ALA in 74.0% and intraoperative neurophysiological monitoring in 94.2%. Complete resection of contrast-enhancing tumour was achieved in 22.5% of WHO grade 4 gliomas, 53.3% of WHO grade 2–3 gliomas and 73.7% of metastases… Median overall survival was 12.7 months in WHO grade 4 glioma and 22.2 months in metastasis, and was not reached in WHO grade 2–3 glioma."
Limitations	19	Discuss limitations of the study, taking into account sources of potential bias or imprecision. Discuss both direction and magnitude of any potential bias	19	**p.19, lines 520–559:** *Dedicated section 4.1 Limitations and Strengths.*<br>**p.19, lines 521–524:** "The study was retrospective, unmatched and non-randomised, and spanned a long period (2011 to 2023) with attendant variability in documentation, so observed outcomes cannot be attributed specifically to N-ioUS given the concurrent use of multiple adjuncts."

	Item No.	Recommendation	Page No.	Relevant text from manuscript
				**p.19, lines 524–528:** "…the figure of 90.4% represents the proportion in which the navigation system used was explicitly recorded rather than a confirmed absence of navigation in the remaining ten cases." *Direction of bias stated: 90.4% understates true navigation use.*<br>**p.19, lines 536–538:** "The metastasis local-control estimate relies on reoperation as a surrogate for recurrence and therefore overestimates true local control." *Direction stated.*<br>**p.19, lines 543–559:** *Further dataset limitations: non-volumetric extent of resection, inferred IDH status, absent MGMT and 1p/19q, no progression-free survival, deficits not classified as temporary or permanent, cause of death unavailable.*
Interpretation	20	Give a cautious overall interpretation of results considering objectives, limitations, multiplicity of analyses, results from similar studies, and other relevant evidence	17, 18, 19	**p.17, lines 446–449:** "Because 40 of 42 grade 4 patients also received 5-ALA, this cohort cannot separate the contributions of N-ioUS and 5-ALA, and no independent or comparative treatment effect can be inferred; the data show only that outcomes appeared broadly similar to the historical 5-ALA cohorts."<br>**p.17, lines 458–459:** "These comparisons are exploratory and uncontrolled and are limited by the small size of some subgroups."<br>**p.18, lines 502–509:** "Brain shift and brain deformation are real, and N-ioUS addresses them directly by restoring an accurate anatomical picture during resection. But in functional-eloquent tumours the anatomical picture is not what limits the operation… the endpoint is a functional boundary, and knowing more precisely where the tumour margin lies does not move that boundary."<br>**p.17–18, lines 420–431 and 487–493:** *Findings set against the same-institution 5-ALA cohorts and against published local control after resection and stereotactic radiosurgery, with the non-comparability of endpoints stated.*
Generalisability	21	Discuss the generalisability (external validity) of the study results	19	**p.19, lines 515–519:** "Finally, ultrasound interpretation is operator-dependent, and in a high-volume unit in which surgeons already have extensive experience of resection under mapping guidance, the incremental orientation that N-ioUS provides might be smaller than it would be in a less experienced setting. Large prospective multicentre trials are therefore needed to fully define the role of N-ioUS in this cohort of patients."<br>**p.19, lines 528–531:** "The cohort was heterogeneous, which reflects real-world practice but limits direct comparison between subgroups."<br>**p.19, lines 538–541:** "The principal strengths are that it examines navigated intraoperative ultrasound within a modern multimodal workflow, that it reflects unselected real-world practice across a broad range of pathologies…"
**Other information**				
Funding	22	Give the source of funding and the role of the funders for the present study and, if applicable, for the original study on which the present article is based	20	**p.20, line 577:** "Funding: This research received no external funding."<br>**p.20, line 587:** "Conflicts of Interest: The authors declare no conflicts of interest."

*Give information separately for cases and controls in case-control studies and, if applicable, for exposed and unexposed groups in cohort and cross-sectional studies.

Note: An Explanation and Elaboration article discusses each checklist item and gives methodological background and published examples of transparent reporting. The STROBE checklist is best used in conjunction with this article (freely available on the Web sites of PLoS Medicine at http://www.plosmedicine.org/, Annals of Internal Medicine at http://www.annals.org/, and Epidemiology at http://www.epidem.com/). Information on the STROBE Initiative is available at www.strobe-statement.org.
